# Achilles Tendon Rupture: Mechanisms of Injury, Principles of Rehabilitation and Return to Play

**DOI:** 10.3390/jfmk5040095

**Published:** 2020-12-17

**Authors:** Domiziano Tarantino, Stefano Palermi, Felice Sirico, Bruno Corrado

**Affiliations:** Department of Public Health, University Federico II of Naples, 80131 Naples, Italy; stefano.palermi@unina.it (S.P.); sirico.felice@gmail.com (F.S.); bruno.corrado@unina.it (B.C.)

**Keywords:** Achilles tendon, tendon rupture, rehabilitation, return to play

## Abstract

The Achilles tendon is the thickest, strongest and largest tendon in the human body, but despite its size and tensile strength, it frequently gets injured. Achilles tendon ruptures (ATRs) mainly occur during sports activities, and their incidence has increased over the last few decades. Achilles tendon tears necessitate a prolonged recovery time, sometimes leaving long-term functional limitations. Treatment options include conservative treatment and surgical repair. There is no consensus on which is the best treatment for ATRs, and their management is still controversial. Limited scientific evidence is available for optimized rehabilitation regimen and on the course of recovery after ATRs. Furthermore, there are no universally accepted outcomes regarding the return to play (RTP) process. Therefore, the aim of this narrative review is to give an insight into the mechanism of injuries of an ATR, related principles of rehabilitation, and RTP.

## 1. Introduction

The Achilles tendon, which is about 12–15 cm long and comprises both the gastrocnemius and the soleus tendons, is the thickest, strongest and largest tendon in the human body. It arises near the middle of the calf and rotates approximately 90 degrees laterally during its course to insert on the posterior aspect of the calcaneal tuberosity [1,2].

Despite its size and tensile strength, Achilles tendon is the most commonly injured tendon in the human body [3,4].

Achilles tendon ruptures (ATRs) occur mainly during sports activities, more frequently in middle-aged men, especially untrained and recreational athletes who play sports occasionally, even though ruptures can occur in younger people [5]. The incidence of ATRs has increased over the last several decades, probably as a result of widespread sports participation [6,7]. Patients with an ATR report sudden and severe pain in the acute phase, and, if left untreated, the injury results in worsened physical function [8].

Achilles tendon tears necessitate a prolonged recovery, leaving a 10% to 30% reduction in functional calf strength and endurance [4,9,10,11,12,13,14], despite increased muscle activity [15,16]. The injury produces long-term limitations [9,15,17,18], and many patients fail to return to sports activities at the same level of performance as before injury [19].

The correct diagnosis of ATR may be missed in up to 25% of patients at initial presentation [20,21,22]. The diagnosis relies on clinical examination, and imaging techniques can be useful in providing additional clinical information. Patients with an ATR usually report a history of pain in the affected leg and the feeling that, at the time of injury, they had been kicked in the posterior aspect of the lower leg or complain of a popping or giving way sensation in their heel after pushing off [23,24]. On clinical examination, diffuse edema and bruising are usually present, and, unless the swelling is severe, a palpable gap may be felt along the course of the tendon, most frequently 2 to 6 cm proximal to the insertion of the tendon [25]. Inspection and palpation should be followed by other tests to confirm the diagnosis, such as the Simmonds (or Thompson) and Matles test and the O’Brien and Copeland tests [23]. Imaging, especially diagnostic ultrasound (that is generally considered the primary imaging method) and magnetic resonance imaging [26,27,28], plays only an adjunct role in the diagnosis and monitoring of ATRs, and it is recommended to rely primarily on clinical examination and evaluation, and to use imaging for ruling out other injuries [28].

After rupture, tendons heal forming scar tissue, and most will never regain the same collagen structure, composition, and organization of healthy tissue [29]. This can lead to a decrease in the mechanical properties of the tissue and increased potential for re-rupture [30].

There is no consensus on which is the best treatment for ATRs, and its management is still controversial. Treatment options include conservative treatment and surgical repair [31]. Since the optimal treatment for acute ATR is continuously debated, recent studies have proposed that the choice of either operative or non-operative treatment may not be as important as rehabilitation [32].

Return to play (RTP) is very important for all athletes who suffered from an ATR, but despite an increased interest in RTP in the last years, there is still a lack of objective criteria for the RTP [33].

The aim of this narrative review is to give an insight into the mechanism of injuries of an ATR, related principles of rehabilitation, and RTP.

## 2. Mechanisms of Injury

Acute ATR usually occurs in its midportion, 2–6 cm proximal from the insertion on the calcaneus. The poor vascularity in the main body of the Achilles tendon may play an important role in the pathogenesis of the rupture [34,35].

In general, there are usually no warning symptoms, and the injury frequently occurs with a distinct ankle trauma. The rupture is generally total; true partial ATRs are very rare [35].

Acute ATRs are classically produced by a single high-load impact (for example, an ATR associated with sudden or violent dorsiflexion of ankle or lunge) [29]. Moreover, an acceleration-deceleration mechanism has been reported in up to 90% of sports-related ATRs [29]. Sporting activity plays a major role in the development of problems with the Achilles tendon, especially when inappropriate training sessions are performed [23,36].

Achilles tendon injuries are common in football, tennis, badminton, and jumping, and have a prevalence in running athletes of 11%. However, 1/3 of patients with this pathology do not practice intensive physical activity [29].

Degenerative changes are the most common histological findings in spontaneous tendon ruptures (such as high vascularity, collagen disorganization and hypercellularity relatively close to the ruptured site) and may lead to reduced tensile strength and a predisposition to rupture [29,37,38].

A reduction in the number and diameter of type I collagen fibers that account for 95% of Achilles tendon collagen [39,40], replaced with larger type III fibers that are produced by ruptured Achilles tendon and that are less resistant to tensile forces [41], are also present [42].

In addition, there are several other factors which play a role in the pathogenesis of ATR, including: gastrocnemius-soleus dysfunction, sub-optimally conditioned musculotendinous unit, age, gender, changes in training pattern, poor technique, previous injuries, footwear, poor tendon vascularity, and various pathologic conditions, such as infectious diseases, neurologic conditions hyperthyroidism, renal insufficiency, diabetes, arteriosclerosis, inflammatory and autoimmune conditions, hyperuricemia, genetically determined collagen abnormalities, and high serum lipid concentration [36,43,44,45,46,47,48,49]. Drugs such as anabolic steroids and fluoroquinolones cause dysplasia of collagen fibrils, which decreases tendon tensile strength and increases the risk of ATR [23,50].

## 3. Principles of Rehabilitation

Because there is conclusive evidence that outcomes after surgical and non-surgical treatment of ATR are comparable and the optimal treatment is continuously debated [32,51,52], methods of rehabilitation are becoming increasingly significant [32,53,54]. Nevertheless, data on the course of the recovery after ATR are still limited, potentially resulting in suboptimal rehabilitation [4].

The lack of available data about psychosocial factors related to outcome, the RTP after ATR treatment and novel imaging techniques, is then reflected on a high rate of re-rupture and complications [51,55,56], unpredictable recovery and RTP [15,19,57,58].

Several patient-related (BMI, nutritional status, comorbidities and athletic status) and injury-related (delay in presentation, injury etiology, gap-size) factors have a possible influence on the recovery and final outcome [55,59,60,61].

Regarding the gap-size, two recent studies aimed to assess if the amount of gap between tendon ends could affect the patient-reported outcome following ATR treated with functional rehabilitation [62,63].

Mubark et al. [62] measured the tendon gap with an ultrasound scan on the initial presentation, then patients were followed for a minimum of 1 year and assessed for Achilles Tendon Rupture Score (ATRS), plantarflexion strength, and re-rupture rate. They found that the outcome following nonoperative functional rehabilitation treatment of rupture Achilles tendon did not correlate with the size of the tendon gap, and the study did not show a statistically significant correlation between the tendon gap size and ATRS at 12 months [62].

Yassin et al. [63], on the contrary, stated that increasing tendon gap (especially if >10 mm), measured using dynamic ultrasound scanning, is associated with poorer patient-reported treatment outcome after ATR functional rehabilitation, as measured by ATRS.

Recent studies suggested that functional rehabilitation and early weightbearing should be preferred over traditional immobilization [32,64,65].

The main findings of the described studies are summarized in Table 1. Systematic reviews have been excluded from this table.

A meta-analysis by Mark-Christensen et al. [32] involving 427 participants, with a total of 211 participants treated with functional rehabilitation and 216 treated with immobilization, showed that there were no statistically significant differences between groups, with a trend favoring functional rehabilitation seen regarding the examined outcomes (such as re-rupture rate, RTP, earlier return to work, increased patient satisfaction, etc).

Aufwerber et al. [66] demonstrated that an accelerated post-operative protocol with immediate loading and ankle motion resulted in better general health and vitality at 6 months, but no differences between the groups were found in the recovery of heel-rise function.

Early rehabilitation after open repair for patients with an ATR was found to be helpful for functional recovery and showed better results in the return to work and the Achilles functional score [67]. Superior outcomes following an accelerated functional rehabilitation protocol with immediate weightbearing in a functional brace, together with early mobilization, were also found after minimal invasive Achilles tendon repair [64].

Early weightbearing in a functional brace was found to provide similar outcomes when compared with traditional plaster casting, resulting to be a safe alternative for patients receiving non-operative treatment of ATR, and to be associated with better early functional outcomes and lower costs [68,69,74].

The concerns rose in the past referred to the risk of re-rupture following a functional rehabilitation protocol have been overcome due to new data that support it, which showed lower re-rupture rates when compared with non-weightbearing and casting [70,71,75]. The lowest re-rupture rates were found in strict functional rehabilitation protocols with full weightbearing in boot immediately at full equinus or at 30° of plantar flexion [76].

Tendon lengthening is another significant problem, usually occurring after ATR [12], but few studies tested how early functional rehabilitation affects tendon elongation [72,77].

Valkering et al. [73] found that functional weightbearing mobilization improved early ankle range of motion without the risk of Achilles tendon elongation and without altering long-term functional outcomes.

A recent prospective cohort study by Aufwerber et al. [72] showed that early functional mobilization with immediate weightbearing and ankle motion, when compared with immobilization in a plaster cast for the first 2 weeks, resulted in more Achilles tendon elongation during the early healing period after surgery (i.e., at 2 weeks after surgery), but at 1 year there was no difference in elongation, also with a trend of less muscle atrophy with an accelerated rehabilitation protocol.

The rate of tendon repair after early mobilization seemed to be significantly improved if compared with continuous immobilization, and resulted in the improved orientation of collagen fibers, improved collagen synthesis, increased number and size of fibrils, increased tendon strength, vascularity, breaking strength, and reduced adhesions and scar formation [78,79,80].

Basic science research showed that mechanical stimulation improves tendon repair, which supports the idea that early mobilization and exercise following ATR may be beneficial [81,82].

Earlier research also showed that the loading of healing tendons leads to essential changes in the biologic process of tendon healing [73,83]. Moreover, early weightbearing could theoretically prevent muscle atrophy, stiffness, adhesions, and deep venous thrombosis (DVT), and has been associated with faster healing and stronger tendons because of improved vascularization and an improved immunologic response [84,85,86].

In any case, some authors found that early controlled ankle motion did not reduce the incidence of DVT when compared to immobilization [87]. In order to effectively minimize the risk of DVT, Aufwerber et al. [66] suggested that patients should be encouraged to load at least 50% of body weight on the injured leg 1 week after surgery.

Therefore, early mobilization and early functional rehabilitation after operative and non-operative treatment of ATRs has been advocated since they lead to new tendon formation and better ultimate functional outcomes, and do not increase post-operative complications [64,88,89,90,91,92].

There is limited available evidence for optimized rehabilitation regimen, and guidelines for initial rehabilitation are limited as well [65,93].

Recently, the rehabilitation regimen after ATR has become more active, and it is characterized by partial or full weightbearing in the first 2 weeks after surgery, and active controlled mobilizations in the first few days after surgery [65].

A study by Frankewycz et al. [93] analyzed 243 protocols for operative and non-operative treatment for ATR provided by 204 orthopedic and trauma surgery institutions throughout Germany. Even if the majority of protocols allowed increased weightbearing over time, a huge variability in rehabilitation after ATR was found [93].

A post-operative protocol described by Maffulli et al. [94] let patients to be discharged the same day of the operation and allowed them to bear weight on the metatarsal heads of the operated leg using elbow crutches as tolerated. An orthopedic physiotherapist should instruct the patient to use crutches. At the time of discharge from the hospital after the operation, all patients were given an appointment for review 2 weeks post-operatively. At 2 weeks, the plaster was removed, and a commercially available removable walker was applied, with five wedges, each 1.2 cm thick, at the heel. Proprioception, active plantar flexion, inversion and eversion exercises were allowed against manual resistance provided by a physiotherapist. One heel wedge was removed every other week, and, at 6 weeks post-operatively, the patients were left free of the cast and referred to physiotherapy for active mobilization. At 10 or 12 weeks post-operatively, patients were assessed as to whether they were able to undertake more vigorous physiotherapy. Further follow-ups at 14 and 18 weeks were arranged. Patients were reviewed during the sixth post-operative month. They were then followed up at 3 months intervals and discharged 12 months after the operation, once they were able to perform at least five toe raises unaided on the operated leg and after they returned to their work or sport.

Patients usually return to their normal sports activity 6 months after the surgery. Time-based guidelines have suggested resumption of non-contact sports 16 weeks after injury and contact sports 20 weeks after injury, but these recommendations are not evidence based [95].

Furthermore, since strength recovery following operative repair of an ATR has been associated with increased ability to return to a previous level of play in patients with higher level athletic activity prior to injury, athletes who desire a return to high-level performance should be informed about the importance of regaining strength and guided toward effective rehabilitation efforts for this purpose [96].

## 4. Return to Play

RTP is crucial for all the athletes who suffered from an ATR. This kind of injury is significant and severely affects the ability of athletes to play at a high level [33]. However, the goal of surgeon and athlete alike is to try to minimize this impact [33]. Different studies have evaluated the rates of RTP in the last few years, showing how the interest in it has been exponentially growing up. There is still a lack of objective criteria for RTP, and the higher the level of sport, the higher the demands, and the pressure on medical professionals to get athletes back to play as quickly as possible [33].

Objective criteria should be used when possible. In the context of Achilles rupture, the Achilles tendon Total Rupture Score (ATRS) has been used widely as an outcome measure [33]. This score is a patient-reported outcome instrument consisting of 10 questions that demonstrate clinical utility for measuring outcomes after ATR [97]. Hansen et al. [98] found out that a patient’s ATRS at 3 months after injury could predict the ability of RTP at 1 year.

The rate of RTP of each selected study is reported in Table 2.

A systematic review and meta-analysis by Zellers et al. [19] based on 85 studies that included a measure for determining RTP reported that the rate of RTP in all studies was 80%, nearly the same rate of RTP (76%) reported in a more recent systematic review by Johns et al. [99].

Trofa et al. [57] examined RTP and performance among professional athletes after AT repair and compared pre- versus post-operative functional outcomes of professional athletes from different major leagues in the United States. They stated that the 30.6% of the professional athletes included in the study with an isolated ATR treated surgically were unable to RTP. They also pointed out that the athletes who returned to play took part in fewer games, had less play time, and performed at a lower level than their preinjury status [57]. However, these functional deficits were seen only at 1 year after surgery compared with matched controls, such that players who return to play can expect to perform at a similar level with healthy controls 2 years post-operatively [57].

These data were later confirmed by Siu et al. [100], who stated that the professional examined basketball players with unilateral ATR reached their post-injury peak performance level at the second season back, and that the post-injury peak performance was significantly worse than the pre-injury level but was similar to matched non-injured players.

Amin et al. [101] followed 18 professional basketball players of the National Basketball Association (NBA) with Achilles tendon repair and took in account several variables (such as age, BMI, player position, etc) and the NBA player efficiency rating (PER) in order to assess the RTP and performance changes. They found that 7 players never returned to play an NBA game, whereas 11 players returned to play one season, with 8 of those players returning for more than two seasons [101]. The PER was reduced more in the first season than in the second season. Given these results, they concluded that the NBA players who returned to play after repair of complete ATRs showed a significant decrease in playing time and performance, with 39% of players never returning to play [101].

Another study by Trofa et al. [102] examined the RTP, playing time, and performance of professional soccer players of the Union of European Football Associations (UEFA) and Major League Soccer (MLS) following ATR. They found that the 70.8% of the selected soccer players were able to return to play, confirming that nearly the 30% of them (29.2%) were unable to return to play, as reported in their other above-mentioned study [57]. In any case, the major difference between the two studies is that, while in the first study professional athletes reached the same pre-injury performance 2 years post-operatively [57], in the present study, soccer players were found to play fewer minutes 2 years post-operatively compared with their baseline as well as playing less at 1 and 2 years post-operatively compared with uninjured matched controls [102]. The reduction in playing time following an Achilles repair has significant implications for professional players and teams [102].

In a recent retrospective observational study by Lerch et al. [103], 5-year return to sport and subjective satisfaction, minimum 1-year functional outcomes, and complications in patients following non-operative treatment of ATR with early weightbearing rehabilitation were assessed. The results of the study highlighted that non-operative treatment for ATR reported good functional outcome and high patient satisfaction [103]. For patients with a high preinjury activity level (such as athletes involved in competitive sports), return to previous sporting level, assessed by the Tegner Activity Scale (TAS), was possible in 67% of the patients compared to >90% of patients with low preinjury activity level (such as workers or people involved in recreational sports) [103].

Finally, a recent study by Grassi et al. [104] evaluated the RTP of 118 professional male soccer players of League 1 and League 2 following acute ATR and surgical repair identified through internet-based injury reports. Only soccer players with injuries who had at least 1 year of follow-up from the search date were included and those who competed for at least two seasons after returning to play, re-ruptures and number of matches played were reported. Of the 118 players, the 96% returned to unrestricted practice and then competition after an average time of 7 and 9 months, respectively [103]. However, 18% did not return to the same level of play within the two seasons following their return, and the 8% sustained a re-rupture within the first two seasons after RTP [104].

## 5. Conclusions

Trauma mechanisms that lead to an ATR are now well understood, classically occurring due to a single high-load impact or an acceleration–deceleration mechanism [1,29]. Degenerative changes are present in spontaneous tendon ruptures and may lead to a predisposition to ATR [29,37,38].

There is conclusive evidence that outcomes after surgical and non-surgical treatment of ATR are comparable [51,52], and recent literature has indicated that functional rehabilitation without surgery can lead to comparable results (patient-reported outcomes and re-rupture) to surgery, without the risk of complications [105]. However, surgical intervention has been noted to provide improved strength compared with functional rehabilitation, and even if this might not be critical for most individuals, for elite athletes it could be career ending; therefore, surgical intervention was recommended for all elite athletes wishing to return to sport after ATR [106].

Methods of rehabilitation are becoming increasingly significant [32,53,54], but there is still limited available evidence for optimized rehabilitation regimen, limited guidelines for the initial rehabilitation [65,93], and limited data on the course of the recovery after ATR and long-term outcomes of athletes [4]. Early weightbearing and early functional rehabilitation after operative and non-operative treatment of ATRs has been advocated since they lead to new tendon formation and better ultimate functional outcomes (such as return to work) [67,77,88,89,90,91,92,107]. Since there is not a universally accepted protocol of early rehabilitation and timing of weightbearing, further studies are needed in the future [92].

There are no universally accepted outcomes regarding the RTP among the selected studies. Some intrinsic and extrinsic factors could potentially affect the RTP. For example, BMI is a modifiable risk factor, which, when lowered, may be associated with less impairment in sports 1 year after an ATR [108].

The mean rate of athletes being unable to RTP among the above-mentioned studies was 29.7 ± 6.738 [19,57,101,102], and athletes who did not return to their previous level of sport was 25.5 ± 7.5 [103,104].

Even if the RTP could be satisfactory without any significant drop-off in performance upon return, for some athletes the ATR could appear to impact their chances of playing professionally in the future [109].

In accordance with the majority of the previously discussed articles, we can state that functional rehabilitation with early weightbearing after an ATR is superior to traditional immobilization. Moreover, more than 70% of athletes are able to return to play after an ATR.

## Figures and Tables

**Table 1:** Post-treatment interventions and findings.

Author	Year	Type of Study	No. of Patients	Treatment	Post-Treatment Intervention	Findings
Aufwerber et al. [66]	2020	RCT	149 (98 EFM, 51 IM)	Surgery	EFM group: dynamic orthosis, WB as tolerated; IM group: below-knee plaster cast, NWB.	Higher scores in general health and vitality at 6 months in the EFM group.
Kim et al. [67]	2017	Case control study (therapeutic)	56 (32 ER, 24 CR)	Surgery	ER group: short leg splint, tolerable WB at 2-week follow-up; CR group: below-knee cast, tolerable WB at 4-week follow-up.	Better results regarding return to work in the ER group; Better Achilles functional score in the ER group.
Costa et al. [68]	2020	RCT	540 (274 FB, 266 IM)	Conservative	FB group: FB with EWB; IM group: plaster cast, EFM with crutches, NWB on injured hindfoot.	Similar outcomes to traditional plaster casting with FB plus EWB; FB safe option for non-operative treatment.
Maempel et al. [69]	2020	RCT	140 (69 FR+EWB, 71 IM)	Conservative	FR+EWB group: walking boot, immediate FWB; IM group: immobilizing cast, 8 weeks of NWB.	FR with EWB safe alternative to traditional IM; Better early functional outcomes with FR.
Hutchinson et al. [70]	2015	Descriptive case series	273 (SMART programme)	Surgery, conservative	SMART group: functional orthoses, EWB, accelerated exercise regime.	Low re-rupture rate; Satisfactory outcomes; Reduced rate of surgical intervention; Reduced healthcare costs.
Aujla et al. [71]	2019	Prospective comparative study	442 (LAMP protocol)	Conservative	LAMP group: 8-week functional dynamic treatment, functional boot with EWB.	Less overall time in the boot; Low complication rates; Similar patient reported outcomes.
Aufwerber et al. [72]	2020	Cohort study	86 (55 EFM, 31 IM)	Surgery	EFM group: dynamic orthosis, FWB; IM group: below-knee plaster cast, NWB.	More elongation at early healing with EFM, but it subsides over time.
Valkering et al. [73]	2017	RCT	56 (27 FWB, 29 IM)	Surgery	FWB group: functional mobilization, functional boot; IM group: below-knee plaster cast, NWB.	Enhanced early healing response with functional WB in the FWB group; Improved early ankle range of motion without risk of elongation in the FWB group.

RCT = randomized controlled trial; EFM = early functional mobilization; IM = immobilization; WB = weightbearing; NWB = non-weightbearing; ER = early rehabilitation; CR = conventional rehabilitation; FB = functional bracing; EWB = early weightbearing; FR = functional rehabilitation; SMART = Swansea Morriston Achilles Rupture Treatment; LAMP = Leicester Achilles Management Protocol; FWB = full weightbearing.

**Table 2:** Rate of RTP.

Author	Year	No. of Patients	Type of Sport	Rate of RTP
Zellers et al. [19]	2016	6506	ND	80%
Johns et al. [99]	2020	333	Football, basketball, baseball, soccer	76%
Trofa et al. [57]	2017	62	Basketball, football, baseball, hockey	69.4%
Siu et al. [100]	2020	12	Basketball	83.3%
Amin et al. [101]	2013	18	Basketball	61%
Trofa et al. [102]	2018	24	Soccer	70.8%
Lerch et al. [103]	2020	89	ND	>70%
Grassi et al. [104]	2020	118	Soccer	96%

ND = not defined.
